# Refined Spherulites of PP Induced by Supercritical N_2_ and Graphite Nanosheet and Foaming Performance

**DOI:** 10.3390/polym15051204

**Published:** 2023-02-27

**Authors:** Ya Liu, Yanjin Guan, Jiqiang Zhai, Lei Zhang, Fengjiao Chen, Jun Lin

**Affiliations:** Key Laboratory for Liquid-Solid Structural Evolution and Processing of Materials, Ministry of Education, Shandong University, Jinan 250061, China

**Keywords:** PP/GN, grain growth rate, secondary nucleation model, foaming behavior

## Abstract

The isothermal crystallization properties of polypropylene/graphite nanosheet (PP/GN) nanocomposites under supercritical N_2_ were systematically studied by a self-made in situ high-pressure microscope system. The results showed that the GN caused irregular lamellar crystals to form within the spherulites due to its effect on heterogeneous nucleation. It was found that the grain growth rate exhibits a decreasing and then increasing trend with the enhancement of N_2_ pressure. Using the secondary nucleation model, the secondary nucleation rate for spherulites of PP/GN nanocomposites was investigated from an energy perspective. The increase in free energy introduced by the desorbed N_2_ is the essential reason for the increase in the secondary nucleation rate. The results from the secondary nucleation model were consistent with those acquired through isothermal crystallization experiments, suggesting that the model can accurately predict the grain growth rate of PP/GN nanocomposites under supercritical N_2_ conditions. Furthermore, these nanocomposites demonstrated good foam behavior under supercritical N_2_.

## 1. Introduction

Many functional lightweight microcellular foams have been prepared based on developed microcellular foaming technology [1,2], which are widely used in industrial applications requiring properties such as sound insulation [3], thermal insulation [4,5], and electromagnetic shielding [6,7]. The crystallization kinetics of composites directly affect cellular structure, which in turn affects the physical properties of the microcellular foam.

Primary nucleation and secondary nucleation together constitute the entire crystallization behavior of the polymer. Primary nucleation is a “from nothing to something” process, i.e., the formation of ordered regions in the disordered phase, which can be characterized by grain density. Secondary nucleation describes the process of continued growth in the nucleus [8] and is usually quantified by the grain growth rate [9,10]. Currently, studies on the crystallization behavior mainly focus on the total crystallization kinetics [11,12,13], without separating the primary and secondary nucleation [14,15]. Numerous scholars have explored the total crystallization kinetics based on the Avrami equation [16], which determines the crystallization behavior through the crystallization rate constant as well as the Avrami index [17,18,19]. However, the crystallinity increases due to further refinement of crystals; in practice, the calculated Avrami index is not an integer [20]. Therefore, the quantitative interpretation of the nucleation and crystal growth patterns by Avrami’s index is not reliable. A secondary nucleation model of the crystal growth frontier was later developed by Lauritzen and Hoffmann and was mainly used to determine the regime transition in crystallization behavior.

The presence of supercritical gas increases the free volume, thus enhancing the mobility of molecular chains and giving the polymer molecules a strong plasticizing effect [21,22]. The improvement of polymer crystallization behavior by high pressure gas is related to its solubility in the polymer [23,24]. In order to have a clearer view of the crystal growth of polymer composites under supercritical fluids, the research cannot remain in the study of the total crystallization kinetics [25,26]. Therefore, it is essential to employ in situ high-pressure visualization devices to characterize the crystallization behavior online. However, the above crystallization kinetic model is not applicable to describe the crystallization behavior under supercritical fluid. The current rapid development of supercritical fluid foaming technology makes it important to develop a secondary nucleation model applicable to describe the crystallization behavior under high pressure gas.

In this paper, supercritical N_2_ is used as the high-pressure medium, which can prepare excellent microcellular foams with dense and fine cell structure. The inexpensive and widely used thermoplastic polypropylene as well as the common graphite nanoflakes were adopted as the research objects. The effects of crystallization behavior and grain morphology of PP/GN nanocomposites under high pressure N_2_ were systematically researched using a homemade in-situ high pressure microscope (HPM-2) system. A secondary nucleation model for the three-phase system was successfully developed. The free energy induced by GN was considered. The inherent mechanism of N_2_ pressure on the crystal growth rate of PP/GN nanocomposites was investigated. Finally, the feasibility of PP/GN nanocomposite foaming was verified by using the mold opening foam injection technique (MOFIM).

## 2. Materials and Methods

### 2.1. Materials and Preparation

**Figure 1:**
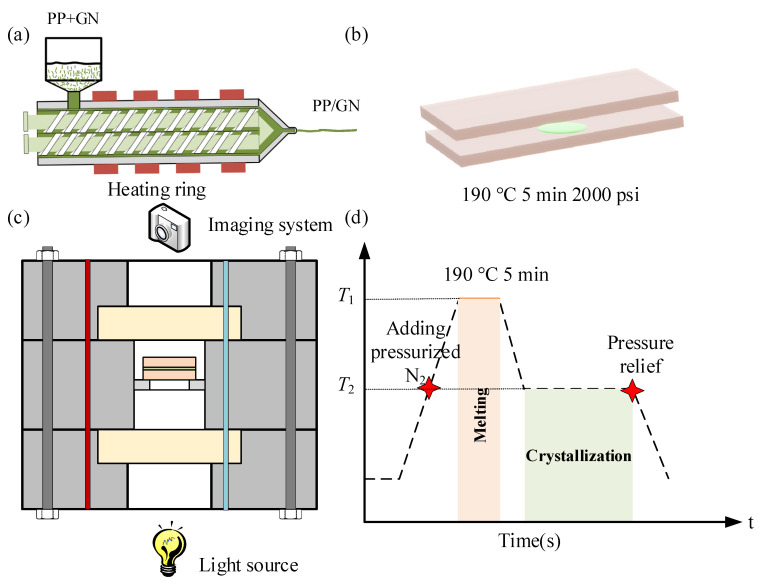
(**a**) Preparation of PP/GN nanocomposites based on twin-screw extruder, (**b**) fabrication of thin films, (**c**) the self developed in-situ visualization system, (**d**) N_2_ and temperature diagram during isothermal crystallization.

Homopolymerized polypropylene (J-150) was supplied by Lotte Chemical Co., which has a density of 0.90 g/cm^3^ and a melt index of 10 g/10 min. The number-average molecular weight (Mn) is approximately 250,000, and its crystallization and melting temperatures are 117.7 °C and 168.7 °C, respectively. The graphite nanoflake (XF011) was prepared by XFNANO Tech. Co., Ltd. (Nanjing, China); its diameter is between 3 and 6 microns, and its thickness is about 40 nm. N_2,_ with a purity of 99.99%, was used as a supercritical fluid. Prior to formal experience, in order to remove moisture, PP was maintained at 80 °C for 4 h. A twin-screw extruder (SJZS-10B, Wuhan, China) was subsequently employed to prepare PP/GN nanocomposites. a homogeneous PP/GN nanocomposites with a GN content of 0.1% were successfully fabricated under the shearing action of the twin screws, which displayed in Figure 1a. A hot press device was adopted to prepare the films for visualization and observation. A small amount of PP/GN was placed between two clean glass sheets to form a sandwich structure, as shown in Figure 1b, which was subsequently placed together on a hot press device and formed by hot pressing at 190 °C and 2000 psi for 5 min. The thickness of thermoforming film is approximately 10 μm.

### 2.2. Online Characterization of Crystallization Behavior

The crystal morphology and crystal growth behavior of PP/GN nanocomposites under different N_2_ pressures were observed by a self-developed in situ high-pressure visualization system, the schematic diagram of which is shown in Figure 1c. Figure 1d displays an N_2_ and temperature treatment diagram during isothermal crystallization. Samples were first held at 190 °C for 5 min to eliminate thermal history. The samples were then cooled to different heat treatment temperatures (T_2_) using alcohol as the cooling medium with a cooling rate of approximately 10 °C/min, at which the nucleation and growth of the crystals were observed. N_2_ was introduced prior to specimen heating and subsequently drained to remove N_2_ after sufficient crystallization.

### 2.3. MOFIM Fabrication Process

The mold opening foam injection molding technology was adopted to fabricate lightweight PP/GN nanocomposite foams. The melt temperatures from the loading port to the injection end were 200 °C, 210 °C, 220 °C, 220 °C, 210 °C, and 200 °C, respectively. However, the mold temperature used in the experiments was 90 °C. The injection rate and shot size to be applied were 100 mm/s and 60 mm^3^, respectively. The packing pressure was held at 40 MPa for 26 s to ensure that the cells formed during the filling process were recompacted and dissolved into the polymer melt. However, the samples were subsequently removed after a cooling time of 40 s.

### 2.4. Characterizations

The Nanomeasure software was used to measure the grain size of the same spherulites at different moments, and the slope of its variation with time is the spherulites growth rate. Grain density was defined as the number of grains in the observation area divided by the area.

After completing the isothermal crystallization behavior of PP/GN nanocomposites on the visualization device, the fully crystallized sample is transferred to the center of the carrier stage. Additionally, the spherical morphology of PP/GN nanocomposites was observed by polarized light microscopy (POM, BX53, Olympus, Tokyo, Japan).

The three-dimensional grain morphology of PP/GN nanocomposites was recorded using a confocal laser microscope (CLSM, LSM 800, Carl Zeiss, Oberkochen, Germany). The spherulite morphology was first observed under the microscope, then switched to scanning mode with the laser turned on to get a clear confocal scan image.

X-ray diffraction (XRD) was employed to determine the crystal structure of PP and PP/GN nanocomposites. The voltage and current used for the tests were 40 kV and 100 mA, respectively. Fourier transform infrared spectroscopy (FTIR) was conducted to investigate the functional groups of PP and PP/GN nanocomposites in the range of 500 cm^−1^ to 4000 cm^−1^.

The cell structure of PP/GN nanocomposite foams was studied with field emission scanning electron microscopy (FE-SEM). The samples were first immersed in liquid nitrogen for 30 min, followed by rapid fracture to keep the section intact. The surface of the sample was sprayed with platinum, and the morphology was observed under SEM.

## 3. Secondary Nucleation Model

The growth process of spherulites includes the formation of nuclei and the growth of grains. The density of spherulites stands for the primary nucleation rate, while the growth rate of spherulites shows the secondary nucleation rate. To quantify the secondary nucleation rate of PP/GN nanocomposites, a secondary nucleation model is usually used to calculate it, which is expressed as follows,

(1) $$I=\frac{nkT}{h}\exp\left(-\frac{\Delta E+\Delta G^{*}+\Delta G_{N}}{kT}\right)$$

where, $\frac{nkT}{h}$ is the prefactor expression, *T* is the isothermal treatment temperature, and *k* and *h* are the Boltzmann constant and Planck’s constant, respectively. *n* stands for the number of kinetic units capable of nucleation, ΔE and $\Delta G^{*}$ represent the diffusion activation energy and the critical nucleation free energy, respectively. $\Delta G_{N}$ is the additional free energy caused by N_2_. The detailed calculation method regarding $\Delta G_{N}$ are discussed in our previous articles [27,28], and can be calculated by,

(2) $$\Delta G_{N}=\Delta G_{m}-\Delta G_{t}+\Delta G_{as}+\Delta G_{aw}$$

where, $\Delta G_{m}$ represents the mixing free energy of the polymer/N_2_ system, $\Delta G_{t}$ is the translational free energy of N_2_, $\Delta G_{as}$ and $\Delta G_{aw}$ stand for the stronger hydrogen-like interaction energy and the relatively weak interaction energy, such as the dispersion force required for the desorption of N_2_ from the polymer homogeneous system, respectively.

The introduction of GN will change the crystallization behavior as well as the crystal morphology of PP materials. Generally speaking, the addition of GN lowers the nucleation barrier of PP crystals, and the entanglement between PP and GN also affects the nucleation of PP molecular chains. Therefore, the free energy variations attributed to GN cannot be neglected, which can be expressed as:

(3) $$\Delta G_{f}=\phi_{3}2\pi D_{t}L_{t}\gamma f\left(\theta \right)$$

where, $\phi_{3}$ is filler volume fraction, $D_{t}$ and $L_{t}$ stand for the diameter and length of snake tube model, respectively. In addition, γ represents the interfacial energy of the matrix and filler, f(θ) is a coefficient considering the interfacial wetting angle.

Finally, after the free energy changes caused by N_2_ and GN are considered, the nucleation rate of the PP/GN/N_2_ system can be determined by [29],

(4) $$I=\frac{nkT}{h}\exp\left(-\frac{\Delta E+\Delta G^{*}+\Delta G_{N}+\Delta G_{f}}{kT}\right)$$

## 4. Results and Discussions

### 4.1. Structure and Morphology

**Figure 2:**
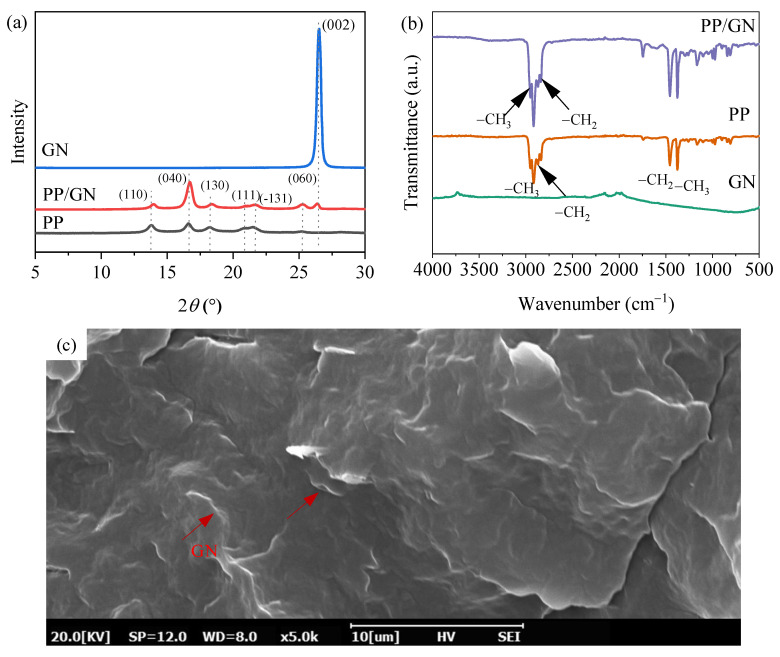
(**a**) XRD patterns of PP and PP/GN nanocomposites, (**b**) FTIR patterns of PP and PP/GN nanocomposites, (**c**) Cross-sectional morphology of PP/GN nanocomposites.

In order to explore the effect of GN on the crystal structure of PP, the XRD spectra of PP, GN, and PP/GN nanocomposites are given in Figure 2a. A strong diffraction peak of GN was found only at 26.52° over the entire test range for the (002) crystal plane, which represents the characteristic π-π stacking [30,31,32]. Pure PP exhibited three strong diffraction peaks at 13.8°, 16.6°, and 18.26°, which correspond to the (110), (040), and (130) crystallographic planes of PP α grains, respectively. Three weaker diffraction peaks were also found at 20.86°, 21.54°, and 25.14°, belonging to the (111), (−131), and (060) crystal planes, respectively. After the incorporation of GN, in addition to the conventional six diffraction peaks, the PP/GN nanocomposites exhibited a diffraction peak corresponding to GN at 26.38. However, no new crystal structures were induced under the current GN content, which also implies that GN is uniformly dispersed in the PP/GN matrix.

The FTIR spectra of PP, GN, and PP/GN nanocomposites are shown in Figure 2b. It can be noticed that four sharp and strong peaks are found near 2917 cm^−1^–2838 cm^−1^, which are characteristic peaks of PP. Among them, the asymmetric stretching vibration peaks of −CH_3_ and −CH_2_ were detected at 2951 cm^−1^ and 2917 cm^−1^, respectively, and the symmetric stretching vibration peaks of −CH_3_ and −CH_2_ were observed at 2871 cm^−1^ and 2838 cm^−1^, respectively. The peaks at 1456 cm^−1^ and 1375 cm^−1^ are due to the bending vibration of −CH_2_ and the symmetric deformation vibration of −CH_3_. No significant detection peaks were found in the FTIR spectrum of GN [33], which further indicates that GN is pure graphite and does not have any oxygen-containing functional groups [34]. As expected, the incorporation of GN does not affect the structure of PP. Figure 2c gives the cross-sectional morphology of the PP/GN nanocomposites. Apparently, GN is uniformly dispersed in the PP matrix. The good dispersion of GN provides the basis for the subsequent analysis of the crystallization behavior.

### 4.2. Crystalline Morphology of PP/GN

**Figure 3:**
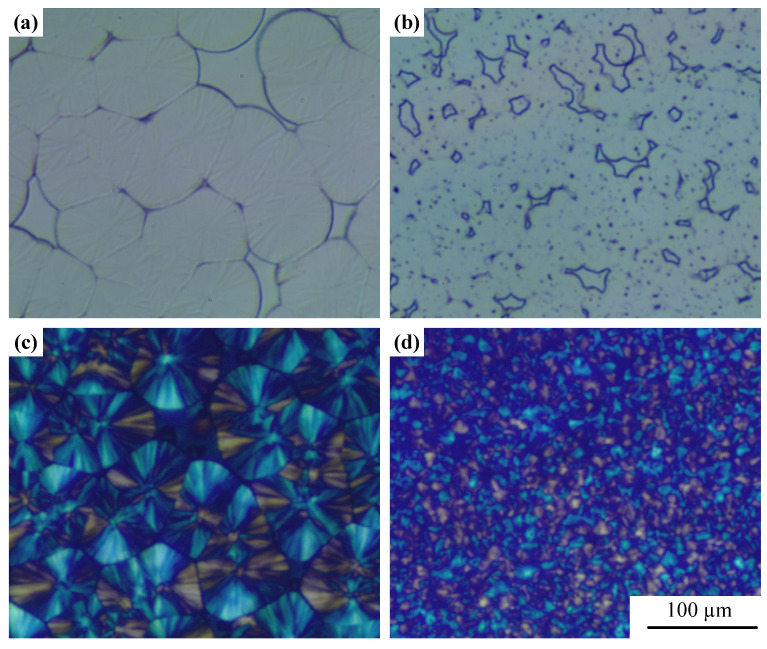
Grain morphology of PP and PP/GN nanocomposite under air and 140 °C, (**a**,**c**) show PP morphology, (**b**,**d**) present PP/GN nanocomposite.

Figure 3 shows the crystallization behavior of PP and PP/GN nanocomposites under air. Larger and sparser spherulites are observed in Figure 3a. After the introduction of GN, the spherulites size is significantly refined, and the spherulites density is greatly increased, as shown in Figure 3b. According to the POM graphs in Figure 3c,d, the PP spherulites possess a clear cross extinction phenomenon and no β-type grains were found [35], which has been verified by the XRD results in the last section. In addition, the refining effect of GN on PP grains is more clearly shown in the POM diagram.

**Figure 4:**
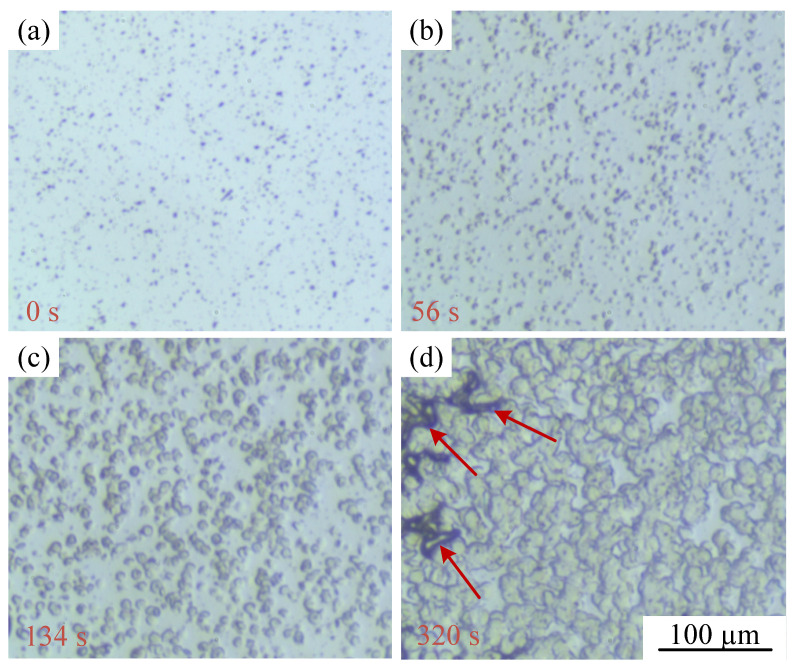
Grain morphology of PP/GN nanocomposite at 140 °C and 13.79 MPa, (**a**) 0 s, (**b**) 56 s, (**c**) 134 s and (**d**) 320 s.

Figure 4 displays optical photographs of PP/GN nanocomposites isothermal crystallization behavior at 140 °C and 13.79 MPa. Firstly, as can be observed from Figure 4a, the distribution of GN within the PP matrix is relatively uniform. Moreover, it can be seen from Figure 4b that many spherulites are nucleated around GN, which fully demonstrates the heterophase nucleation of GN. Due to the facilitative nucleation effect of GN and the reduction of the system’s nucleation energy barrier, GN, as nucleation sites, induce a large number of ordered structures. Consequently, the PP molecular chains on the surface of GN preferentially start the orderly chain arrangement to form spherical crystals first. With the passage of time, spherulites centered on GN were eventually formed, as shown in Figure 4c,d. However, the N_2_ exclusion phenomenon, i.e., where the red arrow in Figure 4d is observed. This is due to the different solubility of N_2_ in the crystalline and amorphous regions. N_2_ can only be dissolved in the amorphous region rather than in the crystalline phase. Above the melting points of PP, PP/GN, and N_2_, they form a homogeneous three-phase system. After dropping to the isothermal treatment temperature, more and more amorphous regions are transformed into crystalline ordered regions as the crystallization process continues to complete. The N_2_ in the decreasing amorphous region is continuously expelled, which is shown in the macroscopic expression as the region shown by the red arrow.

**Figure 5:**
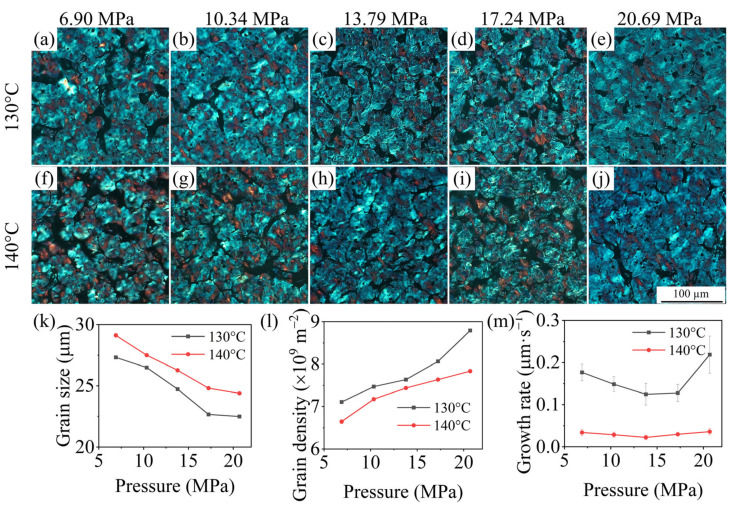
(**a**–**j**) POM photos of PP/GN nanocomposites, evolution of (**k**) Grain size, (**l**) grain density and (**m**) grain growth rate with N_2_ for PP/GN isothermal crystallization at 130 and 140 °C.

Figure 5a–j plot POM images of PP/GN nanocomposites crystallized isothermally at 130 °C and 140 °C under elevated N_2_ pressure. It can be noticed that the cross extinction (Maltese-cross) phenomenon of the grains is not obvious, and the spherulites also behave more coarsely under all experimental conditions. This may be due to the disordered arrangement of the stacked sheet crystals of spherulites, which diminishes their optical anisotropy. It is the heterogeneous nucleation effect of GN that enables the disorderly arrangement of such irregularly shaped and laminated stacked crystals; as shown in Figure 4, the spherulites grow gradually from the GN surface. Moreover, the disorderly stacking of lamellae is further accelerated by the exclusion of N_2_ during grain growth with pressure, which makes this phenomenon more obvious. To quantitatively analyze the effects of T_2_ and N_2_ pressure on the crystallization behavior of PP/GN nanocomposites, the average grain size of spherical crystals was calculated based on the POM diagram, and the grain density as well as the grain growth rate were obtained from the visualization results.

Figure 5k,l provides the dependence of grain size, grain density, and grain growth rate of PP/GN nanocomposites on pressure. PP/GN nanocomposites exhibit the same crystallization behavior across the two T_2_. As the N_2_ pressure increases, the grain size gradually decreases, while the grain density shows the opposite trend. This indicates that although GN has refined the PP grains, supercritical N_2_ still exhibits further refinement of the crystallization behavior. This is due to the plasticizing effect of supercritical N_2_ on PP/GN nanocomposites, which enhances the motility of crystallizable molecules. In addition, the free volume of materials is increased with the addition of N_2_, which induces the rearrangement of molecular chains into a crystal structure with lower free energy and thus easier nucleation. Seeger et al. [36] found that the T_m_ of PP under supercritical N_2_ is slightly augmented with increasing N_2_ pressure. The enhancement in T_m_ implies the formation of more perfect, thicker lamellar crystals and more stable grains [37,38]. These perfect crystals with thicker sheet crystals usually take longer to melt. At the same melting time, the crystalline residues increase with increasing N_2_ pressure due to the melt memory effect. These crystalline residues then become a thermal nucleation site [39,40], which further increase the nucleation density.

Compared to the variation of grain size and grain density with pressure, the grain growth rate demonstrates two different trends within the scope of pressure. As the pressure was lower than 13.79 MPa, the increase in N_2_ pressure showed an inhibitory effect on the grain growth rate, while a further increase in pressure displayed a promotional effect. This interesting phenomenon may be due to the nucleation-limiting effect and the entropy-increasing effect of N_2_ at high pressure. However, the phenomenon is not significant, and the grain growth rate remains lower at higher temperatures, such as 140 °C. This may be due to the lack of self-folding drive for the molecular chains caused by the lower supercooling, which results in a lower growth rate. To further explain this phenomenon from the energy point of view, a secondary nucleation model for a three-phase system was developed.

**Figure 6:**
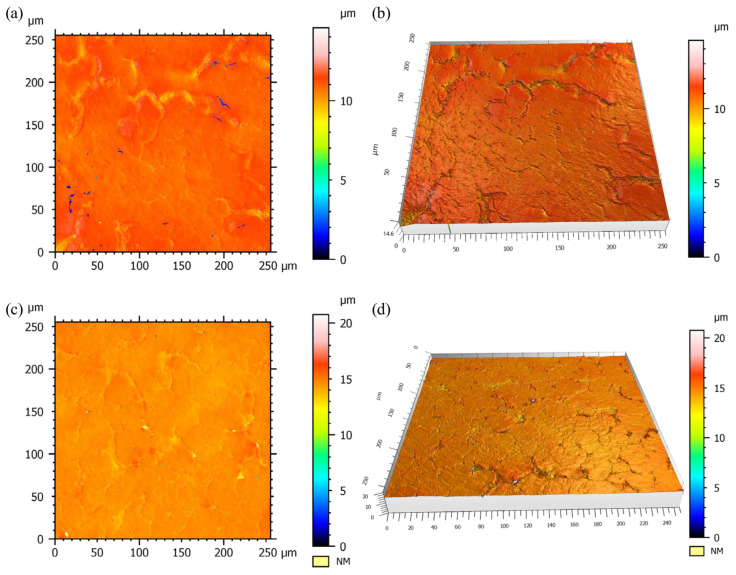
CLSM grain morphology of PP/GN nanocomposite at 13.79 MPa, (**a**) 2D-image and (**b**) 3D-image under 130 °C, (**c**) 2D-image and (**d**) 3D-image under 140 °C.

In order to observe the crystal morphology of PP/GN nanocomposites more clearly, the scanning images using CLSM are given in Figure 6. From the 3D image, it can be seen that the spherical crystals exhibit a radial shape. It is also observed that the *Z*-axis thickness of the spherical crystal that crystallized isothermally at 140 °C is thicker compared to those at 130°C. This indicates that higher layer thicknesses are formed at higher isothermal treatment temperatures. In addition, based on the L-H theory, samples treated at higher isothermal treatment temperatures also have higher melting points. As the crystallization is completed, the PP molecular chains are progressively consumed and N_2_ is continuously discharged. When all molecular chains are depleted, concave molecular dissipation regions as well as the expulsion of N_2_ are found.

### 4.3. Secondary Nucleation Rate of PP/GN Nanocomposite

**Figure 7:**
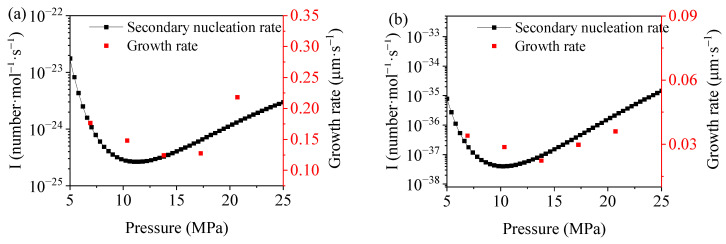
Growth rate calculated by experiments and secondary nucleation rate calculated by model for PP/GN nanocomposite under different temperature, (**a**) 130 °C and (**b**) 140 °C.

Based on the established secondary nucleation model, the mechanism for the effect of high-pressure N_2_ on the grain growth behavior was explored in detail with the addition of 0.1% GN as an example. The experimental results and model predictions regarding the grain growth rate are shown in Figure 7. It can be seen that the calculated results match well with the trends for the experimental results, indicating that the established PP/GN/N_2_ secondary nucleation model can predict the crystallization behavior of PP/GN nanocomposites under supercritical N_2_. Compared with the previously studied pure PP crystallization behavior under supercritical N_2_, there was no significant difference in terms of the growth rate trend, except that it became slower. It still manifests the nucleation-limiting effect of N_2_ at relatively low pressure and the nucleation-promoting effect of N_2_ at higher pressure. As shown in Figure 7a, an interesting phenomenon is that the secondary nucleation rate of PP/GN nanocomposites at 5 MPa is significantly greater than that at 22.5 MPa at 130 °C. However, the difference in secondary nucleation rates between 5 MPa and 22.5 MPa was not considerable at 140 °C. This implies that the re-promotion of N_2_ on the secondary nucleation rate allows the material to grow at the same rate at higher pressures as at lower pressures at higher Tc. This stronger repromotion at high temperatures may be attributed to the relatively high solubility of N_2_ under high temperatures and pressures.

**Figure 8:**
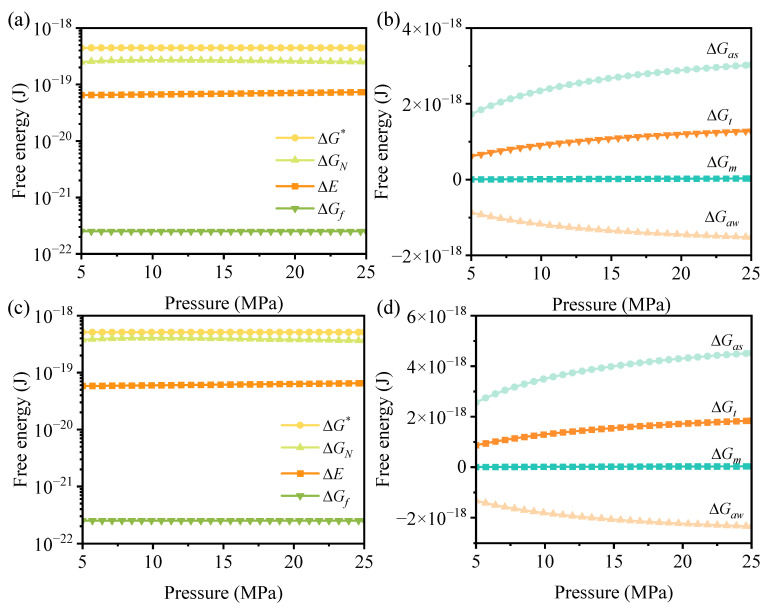
Dependence of $\Delta G_{N}$, $\Delta G_{f}$, ΔE, $\Delta G^{*}$ on N_2_ pressure at (**a**) 130 °C and (**c**) 140 °C, variation of $\Delta G_{as}$, $\Delta G_{t}$, $\Delta G_{m}$, $\Delta G_{aw}$ on N_2_ pressure at (**b**) 130 °C and (**d**) 140 °C.

In order to elucidate the re-promoting effect of supercritical N_2_ on the grain growth rate, Figure 8 shows the variation of each free energy with pressure. The percentage of each free energy at a given pressure is given in Table 1. After the addition of GN, it is still the ΔE and $\Delta G_{N}$ that have a large effect on the secondary nucleation rate. $\Delta G_{f}$, the percentage of free energy caused by GN accounts for only 0.03%, which is due to the relatively small amount of GN added. It is important to note that the positive value of $\Delta G_{f}$ is a resistance to secondary nucleation, which will limit the grain growth rate. This is mainly due to the influence of the interfacial energy existing between GN and PP. For the PP molecular chain to detach itself from the PP/GN/N_2_ three-phase system to nucleate, it needs to conquer not only the influence of N_2_, but also the interfacial energy between PP and GN.

For $\Delta G_{N}$, it shows a tendency to augment and then reduces with increasing pressure, and the proportion for each free energy of $\Delta G_{N}$ is shown in Table 2. It can be found that all $\Delta G_{t}$ rises at each temperature. For example, $\Delta G_{t}$ accounts for only 19.52% at 6 MPa, while 21.8% is recorded with 21.8% as the pressure enhances to 23 MPa with a T_c_ of 130 °C. It is the increase in $\Delta G_{t}$ that impairs the nucleation limiting effect of N_2_ and consequently reveals a re-promotion of the secondary nucleation rate. Therefore, the increase in $\Delta G_{t}$ induced by N_2_ desorbed from the homogeneous system is the underlying reason for the re-promotion effect exhibited by N_2_ at higher pressures, i.e., the entropy-increase induced by N_2_ promotes crystallization.

### 4.4. Foaming Performance of PP/GN Nanocomposite

**Figure 9:**
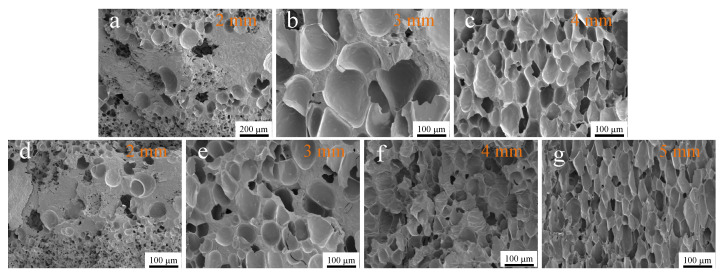
Cell morphology of PP/GN nanocomposite microporous plastic parts prepared by MOFIM technique, PP/0.05%GN nanocomposite foams with opening distance of (**a**) 2 mm, (**b**) 3 mm, (**c**) 4 mm, PP/0.1%GN nanocomposite foams with opening distance of (**d**) 2 mm, (**e**) 3 mm, (**f**) 4 mm, (**g**) 5 mm.

The PP/GN nanocomposite microporous plastic parts were prepared by MOFIM technology, and the foaming feasibility of this material was investigated. Figure 9 demonstrates the cell morphology of PP/GN nanocomposite microporous plastic parts. It can be noticed that the introduced supercritical N_2_ excites the cell structure of PP/GN nanocomposites, and in addition, the addition of GN refines the cell structure of PP materials. At the incorporation of 0.05% GN, the cell size is still large, and the cell density is low. However, the cell size is reduced and the cell structure is denser at the same opening distance after adding 0.1% GN. Furthermore, the 0.1% GN widens the opening distance of PP/GN nanocomposites. This implies that compliant products can be prepared with less raw material, which saves resources.

## 5. Conclusions

The isothermal crystallization behaviors of PP/GN nanocomposites at different treatment temperatures and N_2_ pressures were researched by a self-made in-situ high pressure microscopy system. The PP/GN nanocomposites exhibit a decrease in spherulite size and an increase in spherulite density with enhanced N_2_ pressure within the scope of pressure. The grain growth rate of PP/GN nanocomposite displays a trend of inhibition followed by promotion with rising N_2_ pressure. Based on secondary nucleation model, according to the proportion for the respective energies of nucleation energy and their changes at different N_2_ pressures, it is found that the increased $\Delta G_{t}$ in the homogeneous system under higher pressure N_2_ is the essential reason for the augment secondary nucleation rate, which means that the entropy-increasing effect caused by N_2_ promotes the crystallization. Moreover, PP/GN nanocomposites exhibit good foaming ability at supercritical N_2_.

## Figures and Tables

**Table 1:** Detailed percentage of $\Delta G_{N}$, $\Delta G_{f}$, ΔE, $\Delta G^{*}$ under different pressures.

Temperature (°C)	Pressure (MPa)	$\Delta G^{*}$	$\Delta G_{N}$	ΔE	$\Delta G_{f}$
130	6	57.89	33.6	8.48	0.03
	15	57.03	34.13	8.81	0.03
	23	57.7	32.9	9.37	0.03
140	6	53.38	40.48	6.11	0.03
	15	52.87	40.74	6.36	0.03
	23	54.06	39.1	6.81	0.03

**Table 2:** Detailed percentage of $\Delta G_{as}$, $\Delta G_{t}$, $\Delta G_{m}$, $\Delta G_{aw}$ under different pressures.

Temperature (°C)	Pressure (MPa)	$\Delta G_{as}$	$\Delta G_{aw}$	$\Delta G_{t}$	$\Delta G_{m}$
130	6	53.44	26.82	19.52	0.22
	15	52.23	26.23	21.16	0.38
	23	51.71	25.99	21.8	0.5
140	6	53.53	27.73	18.58	0.16
	15	52.31	27.12	20.3	0.27
	23	51.79	26.88	20.97	0.36

## Data Availability

Data sharing not applicable.
